# Linking household surveys and health facility assessments to estimate intervention coverage for the Lives Saved Tool (*LiST*)

**DOI:** 10.1186/s12889-017-4743-4

**Published:** 2017-11-07

**Authors:** Mufaro Kanyangarara, Victoria B. Chou

**Affiliations:** Department of International Health, Johns Hopkins Bloomberg School of Public Health, 615 N. Wolfe Street, Baltimore, MD 21205 USA

**Keywords:** Service provision assessment (SPA), Service availability and readiness assessment (SARA), Antenatal care (ANC), Lives saved tool (*LiST*)

## Abstract

**Background:**

Calls have been made for improved measurement of coverage for maternal, newborn and child health interventions. Recently, methods linking household and health facility surveys have been used to improve estimation of intervention coverage. However, linking methods rely the availability of household and health facility surveys which are temporally matched. Because nationally representative health facility assessments are not yet routinely conducted in many low and middle income countries, estimates of intervention coverage based on linking methods can be produced for only a subset of countries. Estimates of intervention coverage are a critical input for modelling the health impact of intervention scale-up in the Lives Saved Tool (*LiST*). The purpose of this study was to develop a data-driven approach to estimate coverage for a subset of antenatal care interventions modeled in *LiST*.

**Methods:**

Using a five-step process, estimates of population level coverage for syphilis detection and treatment, case management of diabetes, malaria infection, hypertensive disorders, and pre-eclampsia, were computed by linking household and health facility surveys. Based on data characterizing antenatal care and estimates of coverage derived from the linking approach, predictive models for intervention coverage were developed. Updated estimates of coverage based on the predictive models were compared, first with current default proxies, then with estimates based on the linking approach. Model fit and accuracy were assessed using three measures: the coefficient of determination, Pearson’s correlation coefficient, and the root mean square error (RMSE).

**Results:**

The ability to predict intervention coverage was fairly accurate across all interventions considered. Predictive models accounted for 20–63% of the variance in intervention coverages, and correlation coefficients ranged from 0.5 to 0.83. The predictive model used to estimate coverage of management of pre-eclampsia performed relatively better (RMSE = 0.11) than the model estimating coverage of diabetes case management (RMSE = 0.19).

**Conclusions:**

The new approach to estimate coverage represents an improvement over current default proxies in *LiST*. As the availability of reliable coverage data improves, impact estimates generated by *LiST* will improve. This study underscores the need for continued efforts to improve coverage measurement, while bringing to the fore the importance of health facility assessments as complementary data sources.

**Electronic supplementary material:**

The online version of this article (10.1186/s12889-017-4743-4) contains supplementary material, which is available to authorized users.

## Background

Estimating the impact of scaling up the coverage of maternal, neonatal and child health interventions is important in evaluating progress towards national and global health goals, strategic program planning, and supporting advocacy at local, national, and global levels. The Lives Saved Tool (*LiST*) is a linear deterministic model used to estimate the impact of changes in coverage of key interventions on cause-specific maternal, neonatal, and child mortality in low and middle-income countries [1]. To model the health impact of intervention scale-up in *LiST,* three primary inputs are required: estimates of intervention effectiveness, measures of health and mortality status, and estimates of baseline intervention coverage [2]. Default values for these inputs are provided with the *LiST* software package. The flexible interface allows users to adjust or replace existing values with more recent or appropriate data. For most of the 70+ interventions currently modeled in *LiST,* standard default values for coverage, formally defined as “the proportion of women and children in need of interventions who actually receive them” [3] are derived from large-scale, nationally representative household surveys such as the Demographic and Health Surveys (DHS) [4] and Multiple Indicator Cluster Surveys (MICS) [5].

Standard indicator definitions, sampling designs, and data collection tools are used in the DHS and MICS, to facilitate the tracking of intervention coverage within and between countries, and over time [6]. For example, the DHS and MICS collect data that has been used to track household ownership of insecticide-treated nets (ITNs). However, the utility of large scale household surveys in generating accurate estimates for more complex facility based interventions is limited by respondent recall, and concerns about the validity of self-reported receipt of interventions [6–8]. For example, a woman may be less certain about whether she was screened for syphilis during an ANC visit, but she may more accurately recall having her blood drawn. The validity and reliability of self-reported data represents an ongoing challenge for estimating coverage of interventions along the continuum of care [7–10].

In the absence of reliable and routinely collected data to estimate coverage, default proxies based on expert opinion and panel recommendations have been incorporated into the *LiST* model for some interventions. Coverage of syphilis detection and treatment during ANC, for example, is assumed to vary based on the proportion of women who attended four or more antenatal visits (ANC4+) (Table 1). For case management of conditions such as diabetes, hypertensive disorders, malaria, and pre-eclampsia during pregnancy, default proxies are calculated assuming that 5% of those who attended four or more ANC visits are appropriately screened and managed (Table 1). Although this assumption recognizes that some pregnant women accessing ANC are receiving appropriate and timely case management, the designated cutoff of 5% is a rather arbitrary threshold to apply to all low and middle income countries.

**Table 1:** Definitions and current default proxy values of coverage indicators for selected interventions in the Lives Saved Tool (*LiST*)

Intervention	Indicator definition	Current default proxy applied in Version 5.55 (April 2017)
Antenatal Care (ANC)		
Syphilis detection and treatment	Percent of pregnant women tested for syphilis and given treatment if needed	If ANC4+ is less than 40, 20% of ANC4+ If ANC4+ is between 40% and 75%, 50% of ANC4+ If ANC4+ is between 75% and 95%, 70% of ANC4+ If ANC4+ is 95% or greater,100% of ANC4+
Diabetes case management	Percent of pregnant women screened for diabetes and managed appropriately, if needed	5% of women who attend 4 or more ANC visits (ANC4+) will be appropriately screened and managed
Hypertensive disorders case management	Percent of women receiving detection and appropriate management of moderate to severe hypertension during pregnancy	5% of women who attend 4 or more ANC visits (ANC4+) will be appropriately screened and managed
Malaria case management	Percent of pregnant women experiencing malaria that are appropriately managed	5% of women who attend 4 or more ANC visits (ANC4+) will be appropriately screened and managed
Management of pre-eclampsia with magnesium sulphate	Percent of pregnant women with pre-eclampsia who are treated with intravenous magnesium sulfate	5% of women who attend 4 or more ANC visits (ANC4+) will be appropriately screened and managed

ANC4+ = Percent of women who attend four or more antenatal care visits during their pregnancy

For *LiST*, the accuracy of estimates of intervention coverage at baseline is important as misspecification may bias or skew results (i.e. inaccurate estimation of number of lives saved). As *LiST* is a linear model, the inaccuracy (overestimation or underestimation) of baseline coverage values will not change impact model outputs for scenarios modeling the scale-up of intervention coverage relative to the baseline (e.g. the scale up of newborn outreach interventions by 20% [11]. However, scenarios projecting toward an absolute target (e.g. an increase up to 90% as the target as used in [12] would produce impact estimates which are biased upwards, if proxy estimates for coverage are overly conservative. Conversely, if proxy estimates for coverage are overestimates of true levels of coverage, then the impact of intervention scale up determined by *LiST* would be inadvertently minimized.

Reliable and accurate estimates of intervention coverage are critical inputs to the *LiST* model, and as the availability, validity and reliability of coverage data are strengthened, impact estimates generated by *LiST* will improve. Coverage data may be available in some low and middle income countries, but concerns about the data quality, timeliness and reliability still persist. As part of the broader agenda to end preventable maternal, neonatal and child deaths, calls have been made for improved coverage measurement to track population coverage for life-saving maternal, newborn and child health interventions [3, 13]. Technical work is ongoing to harmonize survey tools and increase the validity and reliability of a core set of indicators used for global monitoring of intervention coverage [6, 9, 14]. One promising approach to improve coverage measurement relies on linking self-reported care-seeking data collected through household surveys to data on service availability and readiness from health facility assessments. There is increasing recognition that this strategy- hereafter referred to as the ‘linking approach’ - may be a feasible option for estimating coverage of interventions not amenable to tracking by household surveys alone [15, 16].

Although the linking of household and health facility surveys represents improved estimates of intervention coverage when no routine coverage data exists, the linking approach depends on the availability and temporal alignment of household and health facility surveys. Although many health facility assessment tools have been developed, nationally representative health facility assessments are not yet routinely conducted and survey data available in many low and middle income countries [17]. For the purposes of *LiST* modeling, it remains crucial to estimate intervention coverage for all low and middle income countries using the available data to inform the process. The objective of this study was to use estimates of intervention coverage derived from the linking approach to guide the development of formulas to calculate new estimates for intervention coverage in *LiST.* For a subset of ANC interventions, we estimated population-level coverage based on the linking approach, then compared these to the existing proxies in the most recent version of the *LiST* model (Spectrum version 5.55, released April 14, 2017). By applying a simple predictive modeling framework, we developed updated estimates for coverage of syphilis detection and treatment, case management of diabetes, hypertensive disorders, malaria infection, and pre-eclampsia. Lastly, we provided recommendations to guide the inclusion of improved estimates of coverage of maternal, newborn and child health interventions in *LiST*. This study underscores the need for continued efforts to improve coverage measurement, and highlights the importance of health facility assessments as valuable data sources.

## Methods

**Fig. 1:**
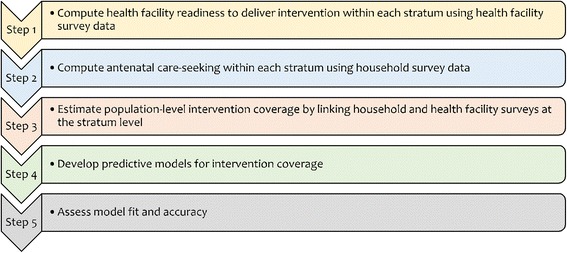
Five step process to develop formulas to update estimates of intervention coverage in the Lives Saved Tool

We used a five step process to develop updated estimates of baseline intervention coverage for syphilis detection and treatment, case management of diabetes, hypertensive disorders, malaria infection, and pre-eclampsia (Fig. 1). First, we used data collected from two large-scale nationally representative health facility assessments, the Service Provision Assessment (SPA) and the Service Availability and Readiness Assessment (SARA), to calculate health facility ‘readiness’ to deliver each intervention. ‘Readiness’ was defined by the availability of the relevant drugs, equipment, supplies, guidelines and trained staff necessary to deliver a specific intervention. Facility-level indicators were summarized at the stratum level as the proportion of health facilities ready to deliver the intervention in that stratum. Strata were defined by health facility type (hospital, health center, health post, etc.), managing authority (public, non-public) and location (rural, urban).

Second, we estimated coverage of ANC4+ within each stratum, using data on care-seeking from the DHS. The woman’s questionnaire of the DHS collects information on pregnancy-related care for the most recent live births occurring within 5 years prior to the survey. To reduce recall bias, this analysis was restricted to reports about ANC received for live births occurring only within the 3 years prior. The analysis was also restricted to the sample of women who reported attending at least four ANC visits, as most ANC interventions we considered require more than one ANC visit to be delivered at sufficient quality to have an impact on the intended health outcomes. Also, ANC4+ coverage is a standard indicator used in *LiST*. Based on the reported source of ANC (managing authority and health facility type) and residence (urban/rural), we computed the distribution of ANC4+ coverage by stratum (health facility type, managing authority and residence).

Third, we computed the product of ANC coverage and health facility readiness for each stratum, then summed across all strata. The resulting estimates represented the proportion of women who attended ANC at least four times and sought care at a health facility ‘ready’ to deliver the specific intervention. Of note, these estimates represent the proportion of women for whom an intervention was available (also referred to ‘availability coverage’) [18], but several factors related to clinical practice could hinder women from actually receiving the intervention [19]. Country-specific coverage values for each intervention were available for 20 SPAs or SARAs conducted in 13 sub-Saharan African countries with a DHS conducted 2 years prior or after the SPA/SARA. The full list of countries where coverage could be estimated based on the linking approach were Benin, Burkina Faso, Democratic Republic of Congo, Ghana, Kenya, Namibia, Rwanda, Senegal, Sierra Leone, Tanzania, Togo, Uganda, and Zimbabwe. Further details on the indicator definitions and exclusion criteria can be found in Additional files 1, 2 and 3.

Fourth, we developed simple predictive models for intervention coverage, with the goal to use these in *LiST* to estimate intervention coverage for all low and middle income countries. The pool of candidate independent variables was drawn from existing DHS data characterizing ANC, specifically the number of ANC visits, timing of ANC visits, and receipt of ANC components: measurement of blood pressure, height and weight, and collection of urine and blood samples. These variables were selected based on both the availability in the DHS and the plausibility of an association with intervention coverage, as they represent elements of ANC essential for the delivery of key ANC interventions. For example, measurement of blood pressure is used to screen pregnant women for hypertensive disorders.

Using the country-level estimates of coverage from the linking approach as the outcomes, we specified fractional logit models, with a logit link and binomial error distribution, to determine variables that predict coverage [20]. To identify factors predictive of intervention coverage, we conducted stepwise regression analysis with exit criteria set at the *p* = 0.1 level. Given the small sample size (*n* = 20), the number of parameters in the predictive models was restricted to a maximum of two (excluding the intercept). Assessing for interaction terms, non-linear relationships, and the inclusion of more independent variables may have strengthened our analysis, but was not conducted due to concerns about the sample size.

Lastly, we considered the fit and accuracy of the predictive models using three metrics: the coefficient of determination (R^2^), Pearson’s correlation coefficient (*ρ*) and the root mean square error (RMSE). A higher coefficient of determination and lower value of RMSE indicate better model fit. Correlations above 0.8 were deemed indicative of strong associations, and those in the 0.5–0.8 range were considered moderate associations.

To assess the impact of different baseline values on estimates of the lives saved in the *LiST* model, we considered the scale-up of coverage of magnesium sulphate for the management of pre-eclampsia to 90%, first using default proxy values, then using coverage estimates computed from the prediction model as baseline values of coverage. The year 2015 was used as the baseline year and the target year was the subsequent year. The coverage of all other interventions in *LiST* was held constant to ensure that only the effect of the specific intervention of interest on mortality was modelled. The number of lives saved were estimated for each of the 13 sub-Saharan Africa countries and the difference in impact produced by *LiST* using the different baseline values compared.

All estimates accounted for the complex survey sampling (cluster design and sampling weights) by using svyset command. All analyses were conducted using STATA (College Station, Texas) version 14.2 and current default proxies used as defaults were based on Spectrum version 5.55 (released April 14, 2017) of *LiST*.

## Results

### Comparison of current default proxies with estimates from the linking approach

**Fig. 2:**
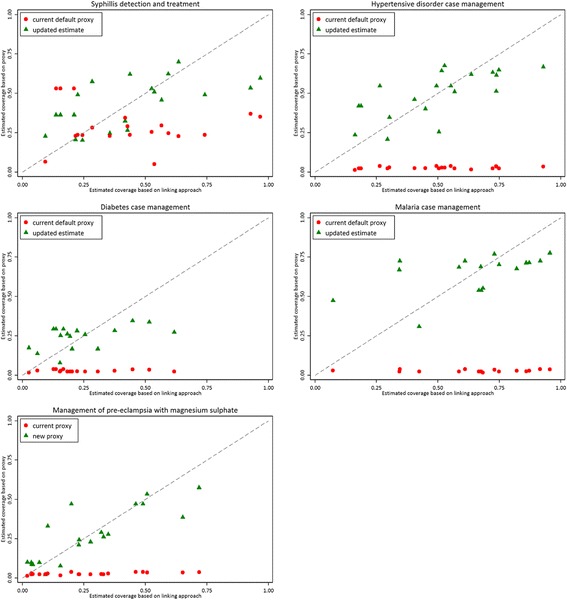
Comparison of current default proxy, linking estimates, and updated estimates of coverage for selected antenatal care interventions

For coverage of case management of diabetes, hypertensive disorders, malaria infection, and pre-eclampsia, comparison of current default proxy values with estimates generated by the linking approach as the “gold standard” indicated that current default proxy values are overly conservative (Fig. 2). Furthermore, the extent to which current default proxy values underestimated coverage varied by intervention, with coverage of case management of malaria infection showing the widest gap, and coverage of case management of diabetes case representing the narrowest. For coverage of syphilis detection and treatment, there was no clear tendency for the existing default proxy values to underestimate or overestimate intervention coverage as estimated by the linking approach. The differences between current default proxy values and estimates from the linking approach ranged from −45% to 34%.

### Predictive models for intervention coverage

From the multivariate analyses, the rate of blood collection and rate of early ANC initiation (<4 months) predicted intervention coverage for syphilis detection and treatment (Table 2). Coverage of hypertensive disorder case management was positively predicted by the rate of blood pressure measurement. The rate of blood collection was a determinant of coverage for both diabetes case management and malaria case management. A higher rate of blood collection together with a higher ANC4+ coverage predicted higher coverage of management of pre-eclampsia.

**Table 2:** Multivariate predictors of ANC intervention coverage

	Syphilis detection and treatment		Case management							
			Diabetes		Hypertensive disorders		Malaria infection		Pre-eclampsia	
	Estimate	p	Estimate	p	Estimate	p	Estimate	p	Estimate	p
Intercept	−1.22	0.17	−3.21	0.001	−1.62	0.001	−1.64	0.10	−6.44	<0.001
Blood sample taken	3.36	0.02	2.61	0.03			2.92	0.02	4.91	0.002
Urine sample taken					2.50	<0.001				
Early ANC enrolment^^^	−5.23	<0.001								
Attended 4 or more ANC visits									2.56	0.05

*ANC* antenatal care, ^^^ Early ANC enrolment was defined as first ANC visit within the first 4 months of pregnancy

### Comparison of updated estimates with estimates from the linking approach

Figure 2 shows the comparison of updated estimates of coverage based on the prediction models versus estimates from the linking approach by intervention. The dashed line represents perfect prediction. The model for coverage of hypertensive disorder case management performed reasonably well with the least scatter around the perfect prediction line. Estimates from models for coverage of syphilis detection and treatment and coverage of malaria case management exhibited more scatter around the perfect prediction line, with several outliers far from the perfect prediction line, and very little agreement.

The measures assessing model fit and accuracy of the five prediction models are summarized in Table 3. Correlation coefficients ranged from 0.5–0.83 indicating moderate to strong agreement between updated estimates and coverage estimates from the linking approach. Independent variables included in predictive models explained between 20 and 63% of the variability in intervention coverage. Overall, the predictive model for case management for pre-eclampsia had relatively good accuracy (RMSE = 0.11). By contrast, the predictive model for coverage of diabetes case management had a poorer model fit (RMSE = 0.19).

**Table 3:** Model performance statistics for models predicting intervention coverage

	R squared	Correlation	Root mean square error
Syphilis detection and treatment	0.38	0.63 (*p* = 0.002)	0.19
Diabetes case management	0.20	0.50 (*p* = 0.04)	0.14
Hypertensive disorder case management	0.47	0.70 (*p* < 0.001)	0.15
Malaria case management	0.28	0.52 (*p* = 0.03)	0.19
Management of pre-eclampsia with magnesium sulphate	0.63	0.83 (*p* < 0.001)	0.11

### Comparison of model outputs using updated estimates and current default proxies as baseline coverage

We assessed difference in impact estimates for the scale-up of coverage of magnesium sulphate for the management of pre-eclampsia to 90% applying the two difference baseline coverage estimates. Given the universally lower default proxy values of coverage, scale up of coverage to 90% in the following year resulted in marginally diminished impact estimates using the updated estimates than default proxy values (Table 4). For Burkina Faso where there was a negligible difference in baseline values (current default: 2% vs updated: 8%), there were similarly negligible differences in maternal lives saved (3.7%) and stillbirths prevented (2.2%). By contrast, for Ghana which represented the widest gap in baseline values (current default: 4% vs linking: 60%), impact estimates dropped to 47% (84/177) and 60% (532/885) of the original maternal and stillbirth impact respectively.

**Table 4:** Comparison of impact estimates for the scale up of coverage of magnesium sulphate for management of pre-eclampsia to 90%

	Baseline coverage (%)			Impact estimates			
				Maternal lives saved		Stillbirths prevented	
	*Current*	*Updated*	*Diff*	*Current*	*Updated*	*Current*	*Updated*
Benin	3	28	25	117	98	497	445
Burkina Faso	2	8	6	273	263	435	425
DRC	2	10	7	2338	2236	2070	2015
Ghana	4	64	60	177	84	885	532
Kenya	3	43	40	804	571	87	70
Namibia	3	50	47	17	11	34	26
Rwanda	2	37	34	124	95	295	249
Senegal	2	26	24	186	158	281	254
Sierra Leone	4	47	43	313	211	278	215
Tanzania	2	29	27	717	594	641	570
Togo	3	33	30	88	71	380	330
Uganda	2	24	22	623	539	1638	1497
Zimbabwe	4	58	54	153	84	486	326

Current = current default proxy value in *LiST*; Updated = updated estimates based on formulas; Diff = absolute difference between current default proxy and updated estimate

## Discussion

We developed formulas to calculate updated estimates for the coverage of syphilis detection and treatment, case management of diabetes, malaria infection, hypertensive disorders, and pre-eclampsia. The updated estimates of coverage offer a few important advantages over the current default proxies in *LiST*. Our approach to coverage estimation was data-driven, making use of country-specific information collected through household and health facility surveys. Health facility assessments represent a rich, yet under-utilized source of information about the performance of health systems. To date, over 48 SPAs and SARAs have been conducted globally, with the majority in sub-Saharan Africa (Additional file 2). Other health facility assessments have been developed, including the Facility Audit of Service Quality (FASQ), Health Facility Census (HFC), and Service Availability Mapping (SAM). As more health facility assessments are conducted and implementation becomes more routine, the geographic coverage and generalizability of findings from health facility assessments will expand. Recently, three SPAs were been implemented outside of sub-Saharan Africa, in Bangladesh (2014), Haiti (2013), and Nepal (2015).

Updated estimates for coverage accounted for the heterogeneity in coverage by intervention not evident in current default proxies, and indicated that current default proxies are most likely conservative. For example, current default proxies for coverage of case management of diabetes, hypertensive disorders, malaria infection, and pre-eclampsia during pregnancy are based on the blanket assumption of that coverage equals 5% of ANC4+ coverage. While the current default proxies are likely an underestimation of true intervention coverage, the resulting bias may not be as apparent when examining mortality reduction as a result of a relative increase in coverage. However, the accuracy of baseline coverage becomes more critical when *LiST* users are interested in projecting the impact of an absolute or universal scale-up. While current default proxies of coverage have been useful in establishing baseline coverage where no routine coverage data exists, as strategies for coverage measurement improve, it is important to continuously update estimates of intervention coverage in *LiST.* More reliable estimates of coverage will inform the setting more appropriate and actionable target goals for coverage, and improve the estimation of projected impact of scaling up intervention coverage in *LiST*.

Our development of updated estimates is based on estimates of coverage derived from linking household and health facility surveys, a methodology that has not been validated, and presents several challenges. Notably, the linking approach relies on the availability of temporally aligned household and health facility surveys. Despite the fairly inclusive definition of temporal alignment (+/− 2 years) used in the present study, our sample size was small, with only 20 health facility surveys from 13 countries in sub-Saharan Africa. As a result, the updated estimates determined in the current study may not truly represent coverage of these interventions in all low and middle income countries. The small sample size also restricted the statistical methods and inclusion of independent variables. As more data become available and constraints due to a small sample size are eased, the inclusion of a broader range of independent variables and use of more sophisticated statistical methods may produce different models and potentially more reliable estimates of country-specific coverage.

Another limitation is related to the assumptions underlying the linking method used. We derived estimates of coverage based on the linking approach, which theoretically determined the proportion of women attending ANC at a health facility deemed ‘ready’ to provide a specific intervention. Readiness was categorized by specifying the minimum conditions necessary to deliver a particular intervention. For example, a health facility was judged to be ready to deliver syphilis detection and treatment if at least one valid syphilis test and drug to treat syphilis were observed in the health facility on the day of assessment. The definitions of health facility readiness for each intervention affected the estimates of coverage obtained by the linking approach. As linking was done at the stratum, and not individual level, large variations in health facility readiness between facilities in the same stratum (health facility type, managing authority and location) will affect the reliability of estimates. Furthermore, factors such as health worker knowledge, supervision and work load will likely reduce the overall proportion of women in need of an intervention who received it [19]. However, it is not possible to determine the extent to which these factors led to biased estimates of coverage.

Ideally, low and middle income countries should strive to establish robust routine health information systems. Unfortunately, given the current challenges it will be necessary to rely on linking household and health facility surveys for the foreseeable future [3, 16, 21]. The combination of two surveys – household surveys to assess care-seeking and health facility assessments to assess the availability and readiness of the health system – will provide more reliable data to track progress towards ending preventable maternal, neonatal and child deaths. The linking approach is a promising strategy, but further research to develop and validate the methods is warranted. Such studies should assess the accuracy and validate estimates of coverage derived from the linking approach. It is important to determine whether linking methods at aggregate levels are sufficiently reliable to determine level of intervention coverage quantitatively and thus, able to be used to track progress over time. Further standardization of health facility assessments and standard definitions of readiness are needed.

## Conclusions

In summary, we updated estimates of coverage for syphilis detection and treatment, case management of diabetes, hypertensive disorders, malaria infection, and pre-eclampsia for use in *LiST*. Work is ongoing to use linking methods to improve estimation of coverage of other interventions along the continuum, where *LiST* currently uses proxy assumptions. While more work is needed to improve coverage measurement for maternal, neonatal and child health interventions more generally, our updated estimates facilitate the establishment of baseline coverage in *LiST.* By balancing the desire for robust estimates of intervention coverage with the limitations of sparse data availability in low and middle income countries, we accomplished the task of improving proxy estimates of intervention coverage, a critical input in *LiST.*

## Additional files

Additional file 1: Table: Indicator definitions. (DOCX 17 kb)

Additional file 2: Table: List of Service Provision Assessments (SPA) and Service Availability and Readiness Assessments (SARA) identified. (DOCX 19 kb)

Additional file 3: Figure: Flow chart of exclusion criteria. (DOCX 28 kb)

## Abbreviations

ANC: Antenatal care
FASQ: Facility Audit of Service Quality
HFC: Health Facility Census
LiST: Lives Saved Tool
RMSE: Root mean square error
SAM: Service Availability Mapping
SARA: Service Availability and Readiness Assessment
SPA: Service provision assessment
