# Supplementary material for: Linking household surveys and health facility assessments to estimate intervention coverage for the Lives Saved Tool (LiST)
Source: BMC Public Health. 2017 Nov 7;17(Suppl 4):780. doi: 10.1186/s12889-017-4743-4 (PMC5688485; doi:10.1186/s12889-017-4743-4)
Supplement: Supplementary file 1 — Table: Indicator definitions. (DOCX 17 kb) [file 12889_2017_4743_MOESM1_ESM.docx]

**Table: Indicator definitions**

| **Indicator** | **Definition of indicators** |
| --- | --- |
| Staff trained in ANC | - At least one staff member trained in at least one aspect of ANC^1^ |
| Guidelines on ANC | - Observed or reported the availability of ANC guidelines |
| Tetanus toxoid vaccine for pregnant women | - Observed at least one valid unexpired unit of tetanus toxoid vaccine |
| Intermittent preventive treatment of malaria in pregnancy | - Observed at least one valid unexpired unit of sulphadoxine/pyrimethamine |
| Syphilis detection in pregnancy | - Observed at least one valid syphilis test^2^ |
| Syphilis detection and treatment in pregnancy | - Observed at least one valid syphilis test^2^ - Observed at least one valid unexpired unit of medicine to treat syphilis^3^ |
| Hypertensive disease case management | - Observed at least one valid dipstick for urine protein OR acetic acid and flame for heating - Observed at least one functioning blood pressure apparatus^4^ - Observed at least one valid unexpired unit of magnesium sulphate |
| Iron supplementation | - Observed at least one valid unexpired unit of iron or iron and folic acid tablets |

^1^ Data on receipt of training of ANC services had varying recall periods ranging from 1-3 years preceding the survey

^2^ Syphilis rapid diagnostic test (RDT), Venereal Disease Research Laboratory (VDRL) test or polymerase chain reaction (PCR) or rapid plasma reagin (RPR)

^3^ Doxycycline, erythromycin, penicillin, or tetracycline

^4^ Automatic or manual with stethoscope
