# Supplementary material for: Linking household surveys and health facility assessments to estimate intervention coverage for the Lives Saved Tool (LiST)
Source: BMC Public Health. 2017 Nov 7;17(Suppl 4):780. doi: 10.1186/s12889-017-4743-4 (PMC5688485; doi:10.1186/s12889-017-4743-4)
Supplement: Supplementary file 2 — Table: List of Service Provision Assessments (SPA) and Service Availability and Readiness Assessments (SARA) identified. (DOCX 19 kb) [file 12889_2017_4743_MOESM2_ESM.docx]

**Table: List of Service Provision Assessments (SPA) and Service Availability and Readiness Assessments (SARA) identified**

|  | **Type of health facility survey** | **Year** | **Status** | **Corresponding household survey** |
| --- | --- | --- | --- | --- |
| Bangladesh | SPA | 2014 | Excluded |  |
| Bangladesh | SPA | 1999–2000 | Excluded |  |
| Benin | SARA | 2013 | Included | DHS 2011-12 |
| Benin | SARA | 2015 | Excluded |  |
| Burkina Faso | SARA | 2012 | Included | DHS 2010 |
| Burkina Faso | SARA | 2014 | Excluded |  |
| Djibouti | SARA | 2014 | Excluded |  |
| DRC | SARA | 2013 | Included | DHS 2013-14 |
| DRC | SARA | 2014 | Included | DHS 2013-14 |
| Egypt | SPA | 2002 | Excluded |  |
| Egypt | SPA | 2004 | Excluded |  |
| Ethiopia | SPA | 2014 | Excluded |  |
| Ghana | SPA | 2002 | Included | DHS 2003 |
| Guatemala | SPA | 1997 | Excluded |  |
| Guyana | SPA | 2004 | Excluded |  |
| Haiti | SPA | 2013 | Excluded |  |
| Indonesia | SARA | 2014 | Excluded |  |
| Kenya | SPA | 1999 | Excluded |  |
| Kenya | SPA | 2004 | Included | DHS 2003 |
| Kenya | SARA | 2013 | Excluded |  |
| Kenya | SPA | 2010 | Included | DHS 2008-09 |
| Malawi | SPA | 2013-14 | Excluded |  |
| Mauritania | SARA | 2013 | Excluded |  |
| Mauritania | SARA | 2015 | Excluded |  |
| Myanmar | SARA | 2014 | Excluded |  |
| Namibia | SPA | 2009 | Included | DHS 2006-07 |
| Niger | SARA | 2015 | Excluded |  |
| Rwanda | SPA | 2001 | Excluded |  |
| Rwanda | SPA | 2007 | Included | DHS 2007-08 |
| Senegal | SPA | 2014 | Included | DHS 2014 |
| Senegal | SPA | 2015 | Excluded |  |
| Senegal | SPA | 2012-2013 | Included | DHS 2012-13 |
| Sierra Leone | SARA | 2011 | Included | DHS 2013 |
| Sierra Leone | SARA | 2012 | Included | DHS 2013 |
| Sierra Leone | SARA | 2013 | Included | DHS 2013 |
| Tanzania | SPA | 2006 | Included | DHS 2004-05 |
| Tanzania | SARA | 2012 | Excluded |  |
| Tanzania | SARA | 2008-09 | Excluded |  |
| Tanzania | SPA | 2014-2015 | Included | DHS 2015-16 |
| Togo | SARA | 2012 | Included | DHS 2013-14 |
| Uganda | SPA | 2007 | Included | DHS 2006 |
| Uganda | SARA | 2012 | Included | DHS 2011 |
| Uganda | SARA | 2013 | Excluded |  |
| Uganda | SARA | 2014 | Excluded |  |
| Zambia | SPA | 2005 | Excluded |  |
| Zambia | SARA | 2010 | Excluded |  |
| Zanzibar | SARA | 2012 | Excluded |  |
| Zimbabwe | SARA | 2014 | Included | DHS 2015 |

DRC: Democratic Republic of Congo, DHS: Demographic and Health surveys
