# Supplementary material for: Linking household surveys and health facility assessments to estimate intervention coverage for the Lives Saved Tool (LiST)
Source: BMC Public Health. 2017 Nov 7;17(Suppl 4):780. doi: 10.1186/s12889-017-4743-4 (PMC5688485; doi:10.1186/s12889-017-4743-4)
Supplement: Supplementary file 3 — Figure: Flow chart of exclusion criteria. (DOCX 28 kb) [file 12889_2017_4743_MOESM3_ESM.docx]

**Figure: Flow chart of exclusion criteria**
